# Asphyxial Death by Ether Inhalation and Plastic-bag Suffocation Instructed by the Press and the Internet

**DOI:** 10.2196/jmir.4.3.e18

**Published:** 2002-10-17

**Authors:** Sotiris Athanaselis, Maria Stefanidou, Nikos Karakoukis, Antonis Koutselinis

**Affiliations:** ^1^Medical SchoolUniversity of AthensDepartment of Forensic Medicine and ToxicologyGreece

Plastic bag suffocation as a method of suicide has been reported before but still remains unusual [1]. On the other hand, accidental deaths are not uncommon among children who play with shopping bags and among adolescents who are solvent abusers. The combination of a plastic bag and an organic solvent is highly unusual [2,3,4]. A case of suicidal death due to a combination ofplastic bag suffocation and diethyl ether inhalation is reported here, based on police and autopsy reports. The remarkable point of this case is that the victim followed instructions from the Internet as well as from a respected international financial magazine.

In February 2002 in Athens, Greece, a 49-year-old male merchant was found by his wife in his office, sitting on his desk with a plastic garbage bag securely fastened around his neck. Inside the bag there was a folded small cleaning towel. Beside him, on the desk was a commercial 500-ml glass bottle of diethyl ether, containing 150 ml of the solvent. The air in the office had an intense smell of ether.

Autopsy showed prominent organ congestion and a remarkable pulmonary edema.

Toxicological analysis of the blood by head-space chromatography revealed the presence of diethyl ether, at a concentration of 127.7 mg/dl, and the absence of any drug or alcohol.

The histopathological examination of the lungs showed a picture of pulmonary edema and prominent congestion. The relevant examination of the liver showed only a congestion of a medium degree.

Apparently, the victim dumped ether on the towel and placed it inside the plastic bag before putting his head in it. He had no history of ether abuse or other substance abuse and he left no suicide note.

The concentration of ether in the blood (127.7 mg/dl) is within the concentrations achieved during surgical anesthesia (50-150 mg/dl) and close to the average concentration for deep surgical anesthesia (120 mg/dl) [5]. This leads us to the conclusion that the death was due to asphyxiation rather than an anesthetic type death. The death was classified as suicide.

According to his wife's testimony, during the previous 10 days the victim was searching the Internet for hours, apparently trying to find a way to commit suicide. A Web site giving thorough advice to people who want to commit suicide [6] was found saved in the "favorites" list on his personal computer. It seems that during the same time the victim was trying to find and read articles on the subject. A related article in the world-famous magazine The Economist [7] was found in his papers on his desk.

The misuse of the Internet - and sometimes of the press, scientific or not - by people that commit suicide must be emphasized. Preventive measures concerning the spread of this kind of information, at a worldwide level, should be taken.
